# Treatment of AML Relapse After Allo-HCT

**DOI:** 10.3389/fonc.2021.812207

**Published:** 2021-12-16

**Authors:** Jonathan A. Webster, Leo Luznik, Ivana Gojo

**Affiliations:** Hematologic Malignancies and Bone Marrow Transplantation Program, Department of Oncology, Johns Hopkins University School of Medicine, Baltimore, MD, United States

**Keywords:** acute myeloid leukemia, allogeneic hematopietic stem cell transplantation, immunotherapy, targeted therapy, donor lymphocyte infusion (DLI)

## Abstract

With advances in allogeneic hematopoietic stem cell transplant (allo-HCT), disease relapse has replaced transplant-related mortality as the primary cause of treatment failure for patients with acute myeloid leukemia (AML). The efficacy of allo-HCT in AML is a consequence of a graft-versus-leukemia (GVL) effect that is mediated by T lymphocytes, and unique mechanisms of immune evasion underlying post-allo-HCT AML relapses have recently been characterized. Relapsed AML following allo-HCT presents a particularly vexing clinical challenge because transplant-related toxicities, such as graft-versus-host (GVHD) and infections, increase the risk of treatment-related morbidity and mortality. In general, the prognosis of relapsed AML following allo-HCT is poor with most patients failing to achieve a subsequent remission and 2-year survival consistently <15%. The two factors that have been found to predict a better prognosis are a longer duration of post-transplant remission prior to relapse and a lower disease burden at the time of relapse. When considered in combination with a patient’s age; co-morbidities; and performance status, these factors can help to inform the appropriate therapy for the treatment of post-transplant relapse. This review discusses the options for the treatment of post-transplant AML relapse with a focus on the options to achieve a subsequent remission and consolidation with cellular immunotherapy, such as a second transplant or donor lymphocyte infusion (DLI). While intensive reinduction therapy and less intensive approaches with hypomethylating agents have long represented the two primary options for the initial treatment of post-transplant relapse, molecularly targeted therapies and immunotherapy are emerging as potential alternative options to achieve remission. Herein, we highlight response and survival outcomes achieved specifically in the post-transplant setting using each of these approaches and discuss how some therapies may overcome the immunologic mechanisms that have been implicated in post-transplant relapse. As long-term survival in post-transplant relapse necessarily involves consolidation with cellular immunotherapy, we present data on the efficacy and toxicity of both DLI and second allo-HCT including when such therapies are integrated with reinduction. Finally, we provide our general approach to the treatment of post-transplant relapse, integrating both novel therapies and our improved understanding of the mechanisms underlying post-transplant relapse.

## Introduction

Allogeneic hematopoietic stem cell transplant (allo-HCT) represents the most effective consolidation strategy to prevent disease relapse for the majority of adult acute myeloid leukemia (AML) patients who achieve remission. Patients with intermediate or poor-risk cytogenetics comprise more than 90% of all newly diagnosed AML patients (1), and myeloablative (MAC) allo-HCT in first complete remission (CR1) leads to improved overall survival (OS) and leukemia-free survival (LFS) when compared to non-transplant approaches in these risk groups (2). Furthermore, a randomized trial in AML and myelodysplastic syndrome (MDS) has demonstrated improved overall survival with MAC allo-HCT compared to reduced-intensity conditioning (RIC) when an HLA-matched donor is used (3). However, the risk of transplant-related mortality (TRM) following MAC makes this approach unsuitable for older patients. Two separate European Group for Blood and Marrow Transplantation (EBMT) analyses demonstrate no benefit to MAC compared to RIC approaches for AML patients aged 40-60 regardless of donor type or patients ≥50 when an unrelated donor is used, as the increased incidence of relapse following RIC is offset by the increased incidence of TRM following MAC, yielding comparable LFS and OS (4, 5). As a consequence of improvements in supportive care that have reduced TRM following MAC and the increased use of RIC, relapse has replaced TRM as the primary cause of treatment failure following allo-HCT for AML and occurs after >30% of transplants (6–8). With more than 3,500 allogeneic transplants performed for AML in the United States alone each year, post-transplant relapse is a common problem (9). The prognosis for patients who relapse following transplant is dismal, as the vast majority do not achieve a subsequent remission, and 2-year OS is consistently <15%. (7, 10). An understanding of the clinical factors influencing survival following post-transplant relapse can help to set expectations and influence the choice of subsequent therapy, which may include intensive induction therapy, low-intensity therapy, targeted therapy, additional cellular immunotherapy, and novel immunotherapy agents. The latter two approaches are of particular interest because the efficacy of allo-HCT in AML is primarily driven by a graft-versus-leukemia (GVL) effect, and there is increasing recognition of unique immunologic mechanisms underlying post-allo-HCT relapse.

Among AML patients who relapse following allo-HCT, the single factor that most consistently predicts subsequent survival is the post-transplant remission duration. In retrospective analyses, the Center for International Bone Marrow Transplant Registry (CIBMTR) demonstrated a 3-year OS of just 4% among AML patients relapsing within 6 months of allo-HCT (10), while the EBMT demonstrated a 2-year OS of 7.5% in patients relapsing within 5 months of RIC allo-HCT for AML (7). Unfortunately, such early relapses are common, as the median time to post-transplant relapse is 7 months, and 43% occur within 6 months (10). The dismal outcomes for early relapse patients suggest that curative outcomes are extremely unlikely, and a palliative approach focused on minimizing therapy toxicity and prioritizing quality of life is often warranted. In contrast, the ability to achieve durable long-term survival following post-transplant relapse increases with the duration of the initial post-transplant remission with the small percentage of patients who relapse 2-3 or 3+ years after transplant having 3-year OS of 26% and 38%, respectively (7, 10). Thus curative outcomes can reasonably be pursued in fit patients with late post-transplant relapses, and an aggressive approach is often merited. Other factors that have been associated with improved survival following post-transplant relapse include: a lower disease burden as assessed by the percentage of bone marrow blasts at relapse, the use of RIC with prior transplant, the absence of acute GVHD prior to or at the time of relapse, the absence of adverse cytogenetics, and age ≤40 years (7, 10). For patients who relapse from 6-24 months post-transplant, these additional factors in combination with the patient’s performance status, co-morbidities, and underlying leukemia biology may help to guide the appropriate approach to subsequent treatment. While the initial goal of therapy in all patients who undergo allo-HCT for AML is cure, a post-transplant relapse should prompt a re-evaluation of this goal with the aforementioned risk factors predicting the likelihood of subsequent durable survival, which can help to inform the choice of a palliative or aggressive approach to subsequent therapy.

There is compelling evidence to support a GVL effect in AML that is mediated by T lymphocytes and correlates with the development of GVHD. The occurrence of both acute and chronic GVHD following transplant has been shown to lead to a reduced risk of post-transplant relapse (11, 12). This suggests correlated post-transplant alloimmune effects that prevent relapse (GVL) and cause side effects (GVHD). Notably, patients who relapse in spite of developing GVHD have poorer survival (7, 10), which is likely a consequence of both the morbidity of GVHD and the ineffectiveness of the GVL effect in these patients. In contrast, an effective GVL effect can be utilized in further therapy, as donor lymphocyte infusion (DLI) alone can produce a subsequent remission in 29% of AML patients who relapse post-transplant and such remissions can be durable when DLI is given after achieving remission with chemotherapy (13, 14). These outcomes with DLI further demonstrate that AML is an immune-responsive disease. T cells are the lymphocytes that are specifically responsible for the GVL effect and the efficacy of DLI, as leukemia patients who received a syngeneic or T-cell depleted allogeneic graft had 3-year relapse incidences of 49% and 35%, respectively, compared to <25% in patients who received a T-cell replete allogeneic graft (11). The ability of AML cells to evade detection or destruction by T lymphocytes could underly post-transplant relapses, and a better understanding of these mechanisms may allow for the safe augmentation of the GVL effect without exacerbating GVHD.

In AML patients who have undergone allo-HCT, two immunologic mechanisms underlying relapse have been characterized. Nearly 50% patients relapsing after transplant demonstrate downregulation of HLA Class II on leukemic blasts irrespective of the number of donor-recipient HLA incompatibilities, which is not seen in AML patients who relapse following chemotherapy alone (15, 16). For AML patients who underwent HLA haploidentical transplant, HLA haplotype loss through acquired uniparental disomy of chromosome 6p occurs in up to 1/3^rd^ of relapsed patients, and donor lymphocytes do not respond to these relapsed leukemic blasts *in vitro* (17, 18). Thus the downregulation of HLA class II molecules or elimination of the non-shared HLA haplotype allows leukemic blasts to evade the GVL effect. A second mechanism that seems to underly AML relapse following allo-HCT is the development of T cell exhaustion. The early detection of severely exhausted bone marrow memory T cells (PD-1^+^Eomes^+^T-bet^-^) predicts relapse, and memory T cells in relapsing patients have increased expression of inhibitory receptors when compared to those who maintain remission at one year (19). These findings suggest that the GVL effect is diminished when allogeneic T cells become exhausted, which can lead to relapse. These two well characterized immunologic mechanisms of relapse (T cell exhaustion and HLA Class II downregulation/haplotype loss) may account for up to 2/3rds of all post-transplant relapses (16), and may have significant implications for the choice of subsequent therapy including immunotherapy.

In the current review, we will outline the current evidence supporting various options for the treatment of relapsed AML following allo-HCT. We will broadly highlight the evidence supporting the use of intensive reinduction with cytotoxic chemotherapy, lower intensity chemotherapy regimens, and targeted therapies including the frequency of achieving remission and long-term survival data. Secondly, we will focus on available immunotherapy strategies for reinduction including DLI, immune checkpoint inhibitors, and other monoclonal antibodies with a focus on how such strategies may overcome known mechanisms of immune evasion and augment the GVL effect. We will additionally discuss the utility of DLI and second allogeneic transplant as consolidation strategies for patients who achieve a second remission. Finally, we will provide our algorithm for how to use these strategies in relapsed AML patients following allo-HCT.

## Intensive Reinduction Therapy

There is a paucity of studies describing the optimal intensive reinduction chemotherapy regimen for post-allo-HCT relapse. Many of the studies describing intensive reinduction chemotherapy regimens for AML have either excluded patients with post-transplant relapse entirely (20), provide no data on outcomes specific to patients who underwent a prior allo-HCT (21–23), or include ≤10 patients with post-transplant relapse (24–27). The dearth of data on intensive reinduction for post-allo-HCT relapse likely reflects the difficulty enrolling such patients on clinical trials due to their myriad complications, such as infections and GVHD. As a consequence of this issue, many of the studies describing intensive reinduction for post-allo-HCT relapse are retrospective; include a variety of reinduction regimens; and often include response endpoints that are assessed after chemotherapy and DLI, making the independent contribution of intensive chemotherapy difficult to assess.

The published literature on intensive reinduction chemotherapy for post-allo-HCT relapse of AML demonstrates reasonable response rates but poor long-term survival in the absence of subsequent cellular therapy in the form of either DLI or a 2^nd^ allo-HCT. Select studies are summarized in **Table 1**, rely largely on cytarabine-based reinduction regimens and demonstrate remission rates ranging from 13-71% with the largest studies showing ≥40% remission rates when chemotherapy is combined with DLI (7, 28–32). While these studies represent a combination of prospective trials and retrospective reviews, larger series suggest that ≥20% of patients receive only supportive care following post-allo-HCT relapse and 33% receive less intensive therapy (7, 10). Thus while intensive reinduction therapy for post-allo-HCT relapse can yield a reasonable response rate, the patients included in these studies are highly selected and the results should not be extrapolated to all patients with post-transplant relapse. The other notable conclusion is that patients who receive intensive reinduction chemotherapy without subsequent DLI or 2^nd^ allo-HCT have dismal long-term outcomes with overall survival of 0-4.4% (7, 30). These poor outcomes partially reflect a selection bias in that patients who fail to respond to reinduction often will not receive subsequent cellular therapy; however, 13-27% of patients in these cohorts did achieve remission and veritably none had durable survival. These outcomes suggest the importance of having a viable option for subsequent cellular therapy when pursuing intensive reinduction therapy.

**Table 1:** Studies including Intensive Chemotherapy for Post-Allo-HCT Relapse.

Authors	Regimens	Subsequent DLI/2nd Transplant	Relapse within 6 months of Prior Transplant	Median Age	N	%CR	ORR	OS
Responses Assessed after Chemotherapy Alone								
Koren-Michowitz et al.	Ara-C + GO	25%/13%	81%	53 (31-63)	16	31%	60%	25% at 1 year
Devillier et al.	HiDAC +/- GO +/- Anthracycline	8%/25%	42%	42	24	71%		33% at 1 year
Schmid et al.	Ara-C + Anthracycline +/-Other, HiDAC +/-Other, Anthracycline + Other	0%/0%	>50%^#^	56 (18-76)^#^	47	27%		4.4% at 2 years
Sauer et al.	HiDAC +/- Anthracycline OR ICE	0%	>50%^$^	52 (17-73)^$^	16	13%		34.4% at 1 year
Responses Assessed after Chemotherapy and DLI								
Motabi et al.	FLAG, FLAG-Ida, FLAG-IM, CLAG, CLAM, MEC, 7+3	56%/7%	58%	52 (18-70)	73	40%	51%	32% at 1 year
Levine et al.*	7+3 (Dauno 30) or Other	100%/3%	55%	42 (2-59)	65	42%		19% at 2 years
Sauer et al.	HiDAC +/- Anthracycline OR ICE	100%	>50%^$^	52 (17-73)^$^	31	48%		29% at 1 year

^#^Median age and time to relapse reflect the full cohort of 776 patients with post-transplant relapse. ^$^Median age and time to relapse reflect the full cohort of 108 patients with post-tramsplant relapse. ^*^Study includes 4 patients with CML and 11 with MDS.

While intensive reinduction chemotherapy for post-allo-HCT relapse of AML can produce a reasonable response rate, the optimal regimen is undefined, and there is a significant risk of complications including non-relapse mortality (NRM) that may increase with additional agents. In prospective randomized trials in relapsed/refractory AML, the addition of a second agent to high-dose cytarabine leads to an increased response rate without yielding a statistically significant improvement in OS (21, 23, 33). While the increased response rate with multi-agent chemotherapy may be beneficial in the post-transplant setting, as it may facilitate more patients receiving subsequent cellular therapy, there is also a risk of increased toxicity, so the use of multi-agent therapy for reinduction should remain an individualized decision in the absence of a clear survival benefit vis-à-vis high-dose cytarabine in the post-transplant setting. Among patients receiving intensive reinduction post-allo-HCT, the early mortality rate is 8-13% (29, 31, 32), but the addition of gemtuzumab to multi-agent chemotherapy led to 15% of patients dying from veno-occlusive disease (VOD) (22). This latter findings highlights the potential risk of additional agents in intensive reinduction for post-allo-HCT relapse. Ultimately, intensive reinduction chemotherapy has a clear role for the treatment of AML relapse following allo-HCT. High-dose cytarabine alone can yield reasonable and durable responses when combined with subsequent cellular therapy, and the use of multi-agent chemotherapy has the potential to increase both response rates and toxicity without a demonstrated survival benefit.

## Lower Intensity Therapy

Hypomethylating agents (HMAs), specifically azacitidine and decitabine, serve as the backbone of lower intensity approaches to reinduction therapy for post-transplant relapse based on their known, modest efficacy in AML, and pre-clinical data suggesting that they can potentially enhance the GVL effect without exacerbating GVHD. In older patients (age ≥65 years) with newly diagnosed AML, azacitidine yields remission in 24.8% of patients and improves OS compared to conventional care regimens (CCR) when censoring for subsequent therapy (34). In addition to its known efficacy, azacitidine is safe and leads to dramatically reduced hospitalizations compared to CCR, which makes it an ideal agent to use in the post-transplant setting. Furthermore, HMAs have been shown to upregulate the expression of tumor cell antigens, HLA class I antigens, and costimulatory molecules on tumor cells (35–38). These changes enhance the ability of allogeneic T cells to both recognize and react to leukemia cells when present, thereby augmenting the GVL effect. HMAs can also increase FOXP3 expression and expand regulatory T cell populations, which animal models suggest may reduce GVHD by suppressing early expansion of alloreactive T cells without inhibiting T cell activation (39–42). Ideally, this means that the GVL effect is preserved in spite of the potential to reduce GVHD. Given the modest clinical efficacy of HMAs in the non-transplant setting and pre-clinical evidence suggesting their particular utility with an alloreactive immune system, numerous studies exploring HMAs in combination with cellular therapy and other agents for the treatment of post-transplant relapse of AML have been completed and select studies are listed in **Table 2**.

**Table 2:** Studies using lower-intensity, HMA-based regimens for Post-Allo-HCT Relapse.

Authors	Treatment	AML/MDS	DLI/2nd Transplant	Median time to relapse post-allo-HCT (mos)	Median Age	N	%CR/CRi	ORR	OS
HMA +/- DLI									
Rautenberg et al.^#^	AZA	60%/40%	70%/11%	4.9 (1-214)	54 (19-71)	151	41%	46%	38% at 2 years
Schroeder et al.	DAC	81%/19%	61%/25%	12.3 (1-87)	56 (21-72)	36	17%	25%	11% at 2 years
Lubbert et al.	AZA (3 days)	92%/8%	65%/27%	8.3 (2-47)	62 (28-75)	26	16%	66%	16% at 2 years
Tessoulin et al.	AZA	61%/39%	39%/3%	3.7 (1.7-37.6)	57 (17-69)	31	14%	35%	Median 5.1 mos
Craddock et al.	AZA	64%/36%	38%/19%	8 (1-71)	NR	181	15%	25%	12.4% at 2 years
HMA + Lenalidomide									
Craddock et al.	AZA/LEN	83%/17%	10%/7%	10 (1-39)	54 (18-73)	29	21%	24%	Median 27 mos in responders, 10 mos in non-responders
HMA + Venetoclax									
Schuler et al.	HMA/VEN	81%/19%	34%/6%	5.7 (1.1-67.8)	54 (31-72)	32	31%	44%	Median 3.7 mos
Joshi et al.^&^	HMA/VEN	66%/34%	0%/3%	9 (2-37)	58 (20-72)	29	28%	38%	Median 2.6 mos

^#^39% of patients in this study were treated for molecular relapse vs. 61% with hematologic relapse. ^&^Three patients received VEN without HMA

### Hypomethylating Agents With DLI

While most large studies describing treatment with HMAs for post-transplant relapse include response assessment following subsequent DLI, it is clear that HMAs can be effective as single agents with particular efficacy as an early intervention for molecular relapse. In a series of ten patients with AML and MDS who relapsed a median of 16 months after transplant (range 0-132 months), Bolaños-Meade et al. demonstrated a CR rate of 60% with azacitidine without unexpected or severe toxicities and survival >1 year in 5 patients of whom only 2 received subsequent DLI (43). While the frequency of responses is notable, there are a number of important caveats: most responses were seen in patients with a remission duration >1 year post-transplant, responses were more common in patients with MDS, and the authors did not rigorously define relapse such that some patients may have only had decreasing donor chimerism at the time of treatment. The RELAZA study demonstrated that the initiation of azacitidine for decreasing post-transplant donor chimerism in AML/MDS led to stabilization or improvement in chimerism in 80% of patients with a few patients sustaining >80% donor chimerism following the cessation of therapy without DLI (44). In this study declining donor chimerism served as a proxy for molecular relapse, and the findings suggest that azacitidine may be more effective when used to treat a low disease burden. This has inspired numerous studies of prophylactic post-transplant azacitidine (45–50), although a randomized trial versus best supportive care recently failed to demonstrate a survival benefit (51). In contrast, a randomized trial of prophylactic low-dose decitabine in combination with recombinant human granulocyte colony-stimulating factor (rhG-CSF) given post-transplant led to a reduction in AML relapses compared to no intervention (52). Thus the evidence does not support prophylactic post-transplant maintenance with HMA alone, but HMAs can serve as an effective treatment for relapses that occur late after transplant or when the disease burden is low and may have a role in combination with other agents as maintenance therapy.

In contrast to the response rates seen with azacitidine for late post-transplant and molecular relapses, response rates to HMAs for morphologic relapse (blasts >5%) are much more modest in spite of the frequent addition of DLI. Four studies of HMAs for morphologic post-transplant relapse in which the majority of patients had relapsed within a year of transplant and received DLI demonstrate remission rates of 14-17% with 2-year OS of 11-16% (53–56). These studies utilized both azacitidine and decitabine including varied dosing schedules of both HMAs without appreciable differences in outcomes, although the retrospective studies suggest that most clinicians choose the conventional dosing schedules for AML/MDS for post-transplant treatment (azacitidine 75 mg/m^2^ daily on days 1-7 or decitabine 20 mg/m^2^ daily on days 1-5). A large German series of patients treated with azacitidine for post-transplant relapse demonstrated that duration of post-transplant remission (>=6 months vs. <6 months) and the type of relapse (molecular vs. morphologic) could be used to stratify patients into three groups with markedly different rates of remission (71% for late molecular relapse vs. 29% for early morphologic relapse) and OS (2-year OS: 64% for late molecular relapse vs. 27% for early morphologic relapse) (57). As the majority of remissions in this study occurred after DLI, it is very difficult to draw firm conclusions about the role azacitidine plays in achieving these outcomes. However, similar to HMA monotherapy, the evidence suggests that HMAs in combination with DLI can be an effective treatment for relapses that occur late after transplant or when the disease burden is low, but the optimal strategy in terms of both HMA dosing and the timing of DLI remains undefined.

### Hypomethylating Agents With Venetoclax

While pre-clinical evidence supports a role for HMAs in enhancing the GVL effect, the potential impact of BCL-2 inhibition on the GVL effect is unclear, but the remarkable efficacy of the BCL-2 inhibitor venetoclax in combination with HMAs for newly diagnosed AML in the elderly suggests that this could be a viable strategy for the treatment of post-transplant relapse. In a murine transplant model, pre-transplant NK cell depletion through BCL-2 inhibition/knock-out leads to improved engraftment, and also improves survival without worsening GVHD in mice inoculated with an MLL-rearranged AML that is insensitive to BCL-2 inhibition (58). However, if post-transplant BCL-2 inhibition robustly depletes alloreactive NK cells, then this could have significant implications in haploidentical transplant, where KIR mismatching of NK cells leads to significant reductions in relapse rates in AML. (59) Future studies of venetoclax in the pre- and post-transplant setting will need to carefully assess its impact on engraftment and relapse, especially following HLA-mismatched transplantation, as it may attenuate the GVL effect that seems to be mediated by NK cells in these transplants. The efficacy of venetoclax in the treatment of newly diagnosed AML in the elderly was demonstrated in a randomized phase III trial in which azacitidine in combination with venetoclax improved both response rates (66.4% vs. 28.3%, p<0.001) and survival (median OS 14.7 vs. 9.6 months, p<0.001) compared to azacitidine alone. While the incidence of febrile neutropenia was significantly higher in the combination therapy group (30% vs. 10%), this did not translate into an increased incidence of early mortality (i.e. within 30 days of treatment initiation) (60). Given the increased risk of infectious complications in the post-transplant setting, one concern is that any disease-control advantage conferred by the addition of venetoclax to HMAs could be offset by increased infectious complications.

In practice the rates of serious and fatal infectious complications have proven much higher in post-transplant and relapsed/refractory than newly diagnosed AML patients treated with HMAs and venetoclax, but the regimen consistently induces remissions post-transplant, even in the absence of DLI. When used in the post-transplant setting, the incidence of fatal infectious complications in patients treated with HMAs and venetoclax is 16%, while the incidence of invasive fungal infections with such regimens more broadly in the relapsed/refractory setting is 19% (61, 62). Thus the use of appropriate, broad antimicrobial prophylaxis is critical in patients treated with HMAs and venetoclax for post-allo-HCT relapse. Venetoclax-containing regimens induce remissions in 28-31% of relapsed AML after allo-HCT without appreciable differences in response among patients who receive DLI, but significantly increased response rates when such regimens are used as the first post-transplant salvage treatment. In these studies, OS was significantly improved in responding patients and those treated for molecular relapse (61, 63). As the combination of HMAs and venetoclax can induce remissions post-transplant, it may serve as a useful bridge to subsequent cellular therapy. However, long-term survival data with this approach is lacking, and the significant risk of fatal infectious complications may nullify the response benefit of combination of therapy in the post-transplant setting. Thus HMAs and venetoclax may be an appropriate lower-intensity therapy for some post-transplant patients, especially in combination with appropriate antimicrobial prophylaxis, but further studies are needed to define its role vis-à-vis the more conventional approach combining HMAs with DLI.

### Hypomethylating Agents With Lenalidomide

Lenalidomide represents another agent with known antileukemic activity and immunomodulatory properties with a recent study demonstrating its efficacy in treating post-transplant relapse in combination with azacitidine. AML patients in remission have been shown to have increased levels of TNFR2+ CD4^+^ T cells and regulatory T cells, leading to reduced IL-2 and IFN-gamma production, which can be reversed by treatment with azacitidine and lenalidomide (64). This could prove particularly relevant in post-transplant relapse patients with downregulation of HLA Class II, as IFN-gamma can rapidly reverse this phenotype *in vitro* (15, 16). However, prior studies of lenalidomide monotherapy in the post-transplant setting have been hampered by the development of GVHD with 43-60% of patients discontinuing for this reason (65, 66). Fortunately, when used in combination with azacitidine for the treatment of post-transplant AML relapse, the incidence of GVHD with lenalidomide was just 10% and 21% of patients achieved a subsequent remission without DLI. Interestingly, there was no evidence that this therapy reversed the exhausted T cell phenotype seen at relapse or led to enhanced cytokine production by T cells in responding patients (67). Further, the low rates of GVHD following lenalidomide treatment seen in this study could be a consequence of the universal use of T cell-depleting GVHD prophylaxis with alemtuzumab or anti-thymocyte globulin in the study population (67). This raises the possibility that an enhanced GVL effect could be seen with azacitidine and lenalidomide among patients who receive T cell-replete grafts, but it also raises the concern for more significant GVHD exacerbations following T cell-replete allo-HCT. Thus additional studies are needed to better define the generalizability of this approach to the more common T cell-replete setting.

## Targeted Therapy

The dramatic expansion of gene sequencing technology over the last two decades has facilitated a better understanding of the important role that somatic gene mutations and gene fusions play in the prognosis of AML and led to the development of molecularly targeted therapies. The most common gene mutations in *de novo* AML include NPM1 (27%), FLT3 (28%), DNMT3A (26%), and IDH1/IDH2 (20%) with roughly 7% being IDH1 and 13% being IDH2 (68, 69). While somatic mutations in the tumor suppressor gene TP53 (8%) and 11q23 rearrangements (4%) are more rare in *de novo* AML, these alterations tend to be enriched in secondary AML (sAML), which is defined as leukemia that has progressed from an antecedent hematologic disorder or is related to prior therapy with cytotoxic agents or radiation (68, 70). As a consequence of the poor prognosis of sAML, transplant is indicated in appropriate patients, but they have a poor post-transplant prognosis due to high rates of relapse and NRM (71, 72). Approved targeted therapies that have been tested in the post-allo-HCT relapse setting include midostaurin and gilteritinib for FLT3+ AML, (73, 74) enasidenib for IDH2+ AML (75), and ivosidenib for IDH1+ AML (76). Additional therapies that have shown efficacy in patients with post-allo-HCT relapse include sorafenib for FLT3+ AML (77, 78), eprenetapopt for TP53-mutated AML (79), and menin inhibitors for NPM1-mutated and MLL-rearranged AML (80, 81). Studies for which specific data on responses to targeted therapy in the post-allo-HCT setting are available are listed in **Table 3**. Interestingly, some targeted therapies yield better responses in the post-allo-HCT setting than in patients without prior transplant, suggesting that alloimmune effects may potentiate their efficacy.

**Table 3:** Studies using Targeted Therapy for Post-Allo-HCT Relapse.

Authors	Agent	Median Age	N^&^	ORR post-transplant	ORR non-transplant	ORR all	OS
FLT3-targeted Therapies							
Perl	Gilteritinib		48	36% (CR)	18%		HR 0.48 (0.27-0.84) vs. chemo for all patients with prior transplant
Metzelder	Sorafenib	45 (14-70)	29	48%	30%	38%	
Bazarbachi	Sorafenib	48 (19-69)	34	39% (CR)			2-year OS 38% vs. 9% for controls-->HR 0.44, p=0.001
IDH1-targeted Therapy							
DiNardo	Ivosidenib		36			42%^	
IDH2-targeted Therapy							
Stein	Enasidenib		29	35%	40%	39%	

^&^Refers to the number of post-allo-HCT patients included in the study. ^The authors note no difference in response rate based on pre-treatment characteristics of which prior allo-HCT was included.

### FLT3 Inhibitors

While not an approved therapy for FLT3-mutated AML, the pankinase inhibitor sorafenib is a potent FLT3 inhibitor and the first targeted therapy to prove effective for post-allo-HCT relapse of AML (82). A study of FLT3-ITD AML patients who had relapsed a median of just 2.8 months post-transplant demonstrated a 39% remission rate in those treated with sorafenib with dramatically improved survival compared to a control group that did not receive sorafenib salvage, and a 2-year OS of 38% among those treated with sorafenib of whom 33% and 13% received subsequent DLI or a second allo-HCT, respectively (77). While the poor results of the control group are difficult to interpret given the potential biases that may have led them not to receive sorafenib in this retrospective study, it is clear that sorafenib can lead to durable responses for post-allo-HCT relapse including those with early relapse. In a separate study of relapsed/refractory FLT3-ITD AML, sorafenib led to high response rates with resistance developing later in patients with a history of allo-HCT, and the only durable responses (i.e. >12 months) seen in post-transplant patients (78). Notably, sorafenib has been shown to increase IL-15 production in mutant FLT3-ITD leukemia cells, increase IFN-γ production, and lead to metabolic reprograming of leukemia reactive T cells among relapsed patients post-allo-HCT who respond to treatment (83). The latter finding may be particularly relevant when there is underlying HLA Class II downregulation, as IFN-γ can reverse this process (15). Sorafenib has also been combined with azacitidine in a prospective trial for the treatment of relapsed/refractory FLT3-ITD^+^ AML with a response rate of 46%, but only seven patients in this study had received a prior allo-HCT (84) which precludes any conclusions about the specific efficacy of this combination in the post-transplant setting. Thus while prospective trial data supporting the use of sorafenib for post-allo-HCT relapse of FLT3-ITD^+^ AML is lacking, there is retrospective clinical data to support its use as monotherapy.

In contrast to sorafenib, the FLT3 inhibitors midostaurin and gilteritinib have both been investigated in prospective trials for relapsed/refractory FLT3-mutated AML that enrolled post-allo-HCT relapse patients. Midostaurin, which is approved for use in patients with newly diagnosed FLT3-mutated AML based on the RATIFY trial (85), in combination with azacitidine was investigated in a phase I/II trial in newly diagnosed, unfit and relapsed/refractory AML that enrolled primarily patients with FLT3 mutations (74%), and demonstrated a dramatically reduced response duration (median 6 weeks) in patients who had received a prior allo-HCT compared to those who had not (74). In spite of the heterogeneity of the patient population in this study, including patients who had received no prior treatment and those without FLT3 mutations, the results suggest that the combination of midostaurin and azacitidine is not effective for the treatment of post-allo-HCT relapse of FLT3-mutated AML. In contrast, the phase III ADMIRAL trial, which randomized relapsed/refractory FLT3-mutated AML patients to gilteritinib or chemotherapy salvage, demonstrated a dramatically improved response rate among gilteritinib-treated patients who had previously undergone allo-HCT (36% vs. 18% for non-transplant patients) and improved survival among post-transplant patients randomized to gilteritinib versus chemotherapy (73). The improved responses among patients with a prior allo-HCT suggests that alloimmune effects may also potentiate the activity of gilteritinib. The compelling response and survival data from the ADMIRAL trial makes FLT3-mutated AML the rare scenario in which the optimal initial therapy for post-transplant relapse is data driven. However, additional data are needed to better understand the necessity of subsequent cellular therapy to facilitate durable survival, and whether there is a role for combining gilteritinib with either intensive therapy, such as high-dose cytarabine, or hypomethylating agents.

### IDH1/2 Inhibitors

After FLT3 mutations, IDH1 and IDH2 mutations represent the next two most common types of gene mutations in AML for which there are approved, targeted therapies, and there is evidence to support their use for the treatment of post-allo-HCT relapse. In a trial in relapsed/refractory IDH1-mutated AML that treated 36 post-transplant patients at the FDA-approved dose, ivosidenib produced an overall response rate (CR, CRi, CRp, or bone marrow CR) of 41.6% without any differences in response noted based on most baseline clinical characteristics, which included prior allo-HCT (76). Further data from this trial may help to elucidate the durability of responses with ivosidenib in the post-allo-HCT setting, although the overall response rate compares favorably with most studies of intensive and lower-intensity salvage therapies following allo-HCT. In a similar trial of enasidenib for IDH2-mutated, relapsed/refractory AML that enrolled 29 patients following allo-HCT, the overall response rate (CR, CRi, CRp, or bone marrow CR) among post-transplant patients was 34.5% compared to 39.5% among patients without a history of transplant (86). As with ivosidenib, additional studies are needed to better understand the durability of responses to enasidenib in the post-transplant setting including the need for further cellular therapy in responding patients. One appealing aspect of ivosidenib and enasidenib use in the post-transplant setting is that their toxicity profile generally compares favorably with intensive chemotherapy, except for the more frequent occurrence of differentiation syndrome (75, 76). Thus targeted therapy for IDH1 and IDH2-mutated AML that has relapsed following allo-HCT represents an effective and potentially less toxic alternative to conventional chemotherapy to achieve subsequent remission.

### TP53-Targeted Therapy

Mutations in the tumor suppressor gene TP53 are frequent in AML patients with adverse cytogenetics and confer an extremely poor prognosis even with allo-HCT with subsequent relapse rates of 60% (87). However, the tumor suppressor function of TP53 can be restored with molecularly targeted therapy. (88) APR-246, now known as eprenetapopt, represents one such therapy, and a patient with relapsed TP53-mutated AML following allo-HCT achieved a complete response following treatment with this agent in combination with sorafenib and hydroxyurea (79). Unfortunately, the combination of therapies that this patient received makes it difficult to isolate the role eprenetapopt played in achieving remission. While two large trials of eprenetapopt in combination with azacitidine for newly diagnosed TP53-mutated myeloid malignancies demonstrated promising results (89, 90), a subsequent randomized phase III trial versus azacitidine monotherapy failed to show a survival benefit (91). Eprenetapopt has also been studied as a prophylactic post-transplant maintenance therapy in combination with azacitidine for patients with TP53-mutated AML and MDS leading a 1-year relapse-free survival of 58% (92). Given the dismal prognosis of TP53-mutated myeloid malignancies following transplant, this result is promising, but a randomized trial versus azacitidine is needed to establish the contribution of eprenetapopt to the durable post-transplant remissions seen in this study. Ultimately, evidence that eprenetapopt is useful as post-transplant prophylaxis is likely to suggest that it may also have a role for the treatment of post-transplant relapse.

### Menin Inhibitors

Breakage of the KMT2A gene, commonly known as the MLL gene and located at 11q23, leads to a poor prognosis AML with relapse rates of 35% following allo-HCT in adult patients (93), while AML with an isolated mutation of NPM1 has an incidence of post-allo-HCT relapse of 12-22% when transplant is performed in first remission (94). KMT2A-rearranged and NPM1-mutated AML are linked by highly upregulated HOX expression, and menin inhibitors lead to cellular differentiation of leukemic cells in both types of AML both *in vitro* and *in vivo*. (95). This is similar to the cellular differentiation that is seen with IDH inhibitors in IDH-mutated AML. Two ongoing trials of menin inhibitors (SNDX-5613 and KO-539) in relapsed/refractory KMT2A-rearranged and NPM1-mutated acute leukemias including patients with a history of allo-HCT are currently enrolling with preliminary results demonstrating their efficacy in producing remissions (80, 81). At the moment, there is no specific data on the efficacy of these agents in post-allo-HCT patients, but they have demonstrated a favorable safety profile to date that suggests the potential for menin inhibitors to achieve similar success for post-transplant relapse in KMT2A-rearranged and NPM1-mutated AML as FLT3 and IDH inhibitors in their respective subtypes.

## Immunotherapy

Given that the GVL effect in AML seems to be primarily mediated by T lymphocytes (11). there is substantial interest in T-cell-directed therapies to combat post-allo-HCT relapse. While post-transplant immunosuppression is critical for the prevention of GVHD, its withdrawal in relapsed patients without evidence of GVHD can unleash alloreactive T cells and even produce remissions in patients with morphologic relapse with low blast counts (<10%) (96). Other T cell-based therapies include DLI, immune checkpoint inhibitors, and dual affinity retargeting antibodies (DARTs), among others. However, stimulating alloreactive T cells post-transplant also dramatically increases the risk of GVHD, which greatly increases the potential morbidity of these therapies. A second allogeneic transplant, which relies on a combination of the GVL effect and conditioning chemotherapy, can also produce prolonged responses, both with and without antecedent chemotherapy to induce remission. One potential intervention that can help to maximize the GVL effect and reduce the post-allo-HCT relapse risk is the use of mobilized peripheral blood stem cells as opposed to a bone marrow graft (97, 98). However, as with other interventions that augment the GVL effect, the use of peripheral blood grafts leads to an increased risk of both acute and chronic GVHD (97, 98). As KIR mismatching has been shown to prevent AML relapse following haploidentical transplantation (59), haploidentical NK cell-based therapy is emerging as another cellular therapy option that may augment the GVL effect. Studies for which there are specific response and outcomes data for the use of immunotherapy for post-allo-HCT relapse of AML are highlighted in **Table 4**.

**Table 4:** Studies using Immunotherapies for Post-Allo-HCT Relapse.

Authors	Therapy	DLI/2nd Transplant	Median time to relapse post-allo-HCT (mos)	Median Age	N	%CR/CRi	ORR	OS
DLI								
Kolb et al.	DLI	100%	7.9 (4.3-33.6)	37 (4-54)	23	22%		Median 248 days in AML/MDS
Schmid et al.	DLI	100%/8%	5.5 (0.1-55)	39 (16-65)	171	35%	38%	2-year OS 20% with DLI
Kharfan-Dabaja et al.	DLI	100%/18%	7.0 (0.7-129)	49 (19-75)	281	24%		5-year OS 15% (10-19%)
Immune Checkpoint Inhibitors								
Davids et al.	Ipilimumab				12	33%	42%	
Davids et al.	Nivolumab				10	0%	0%	
Haploidentical NK Cell Infusion								
Shaffer et al.	NK cell infusion		3.5 (1-94)	19 (1.9-55.9)	8	25%	38%	Median 12.9 months (0.8-65.3 months)
2nd Allogeneic Transplant								
Kharfan-Dabaja et al.	2nd Transplant (MUD)	NR/100%	15 (6-31)	46 (35-58)	320			2 year OS 31% (26-37%)
Kharfan-Dabaja et al.	2nd Transplant (Haplo)	NR/100%	11 (5-25)	44(33-53)	135			2-year OS 29% (20-39%)
Kharfan-Dabaja et al.	2nd Transplant	1%/100%	11.6 (1-152)	43 (18-67)	137	39%		5-year OS 19% (12-25%)

### Withdrawal of Immunosuppression

For AML patients, discontinuation of post-transplant immunosuppression can prevent impending relapse and even induce remission in some cases of morphologic relapse. Leukemia and MDS patients with serially decreasing levels of donor chimerism post-transplant are at significantly increased risk of relapse compared to patients with full donor chimerism and rising levels of donor chimerism (99). This suggests that interventions that reverse declining chimerism may prevent subsequent relapse. In a cohort of 26 patients with hematologic malignancies with incomplete donor chimerism following allo-HCT, 27% achieved full donor chimerism following the early withdrawal of immunosuppression with the majority remaining in durable remission (≥31 months) (100). Among 45 AML and MDS with morphologic relapse post-transplant in the absence of GVHD, the withdrawal of immunosuppression led to complete remission in 6.6%, all of whom had less than 10% blasts in the marrow at relapse (96). While the withdrawal of immunosuppression for post-transplant relapse increases the risk of GVHD, it can yield remission in a limited subset of cases and may also potentiate the GVL effect of subsequent therapy. Thus for relapsed post-transplant patients without GVHD, withdrawal of immunosuppression should generally be the first intervention.

### DLI

Following the withdrawal of immunosuppression for relapsed post-allo-HCT patients without GVHD, DLI can induce remissions, but durable responses are seen only in patients who achieve remission before DLI and those with a favorable karyotype. In a cohort of 171 AML patients who received DLI for post-allo-HCT relapse, 35% achieved remission with 43% and 46% of evaluable patients developing acute and chronic GVHD, respectively (14). This highlights the significant morbidity that is caused by DLI, which makes it essential to carefully select patients who are likely to benefit prior to initiating therapy. In this cohort of patients who universally received DLI for post-transplant relapse, three prognostic groups were defined with patients in remission at the time of DLI and/or with a favorable karyotype having a 2-year overall survival of 56% compared to 21% and 9% in female patients who were not in remission at the time of DLI but had a low tumor burden (<35% blasts) at relapse and all remaining patients, respectively (14). While durable responses are achieved even among patients with active disease, this must be weighed against the risk of morbidity from GVHD. Furthermore, the recently characterized immunoediting capabilities of post-transplant AML with downregulation of HLA Class II genes or loss of haplotype raises the possibility that DLI could be ineffective in relapses involving this underlying mechanism of immune evasion (15–17). If studies clearly demonstrate the ineffectiveness of DLI in this setting, then it will be critical to develop standardized assays to screen for this mechanism, as its presence may necessitate alternative therapy to achieve a durable remission. Finally, there is also no data to support the optimal salvage regimen to achieve remission prior to DLI such that the choice and intensity of chemotherapy necessarily depends on the patient’s performance status, co-morbidities, and the presence of targetable gene mutations.

### Checkpoint Inhibitors

A second immunologic mechanism that seems to underly post-transplant relapse is the development of T cell exhaustion with the increased expression of immune checkpoints (17). This raises the possibility that immune checkpoint blockade could be a useful treatment approach for post-transplant relapse of AML. In a study including 12 AML patients with post-transplant relapse, blockade of cytotoxic T-lymphocyte-associated protein 4 (CTLA-4) with ipilimumab produced a response rate of 42% including complete remission in 4 patients with extramedullary leukemia. A total of 22 post-transplant patients received the maximum tolerated dose of 10 mg/kg of ipilimumab with three patients developing GVHD that precluded further treatment on the study and three patients developing immune-related adverse events (immune thrombocytopenia, colitis, and pneumonitis) of whom two patients were able to resume therapy following treatment with steroids (101). While the side effects of post-transplant ipilimumab are significant, its unique efficacy in patients with extramedullary relapse may justify its use in this scenario. A separate study of PD-1 blockade with nivolumab for post-allo-HCT relapse enrolled 10 patients with AML of whom none had an objective response to treatment, and nivolumab led to dose limiting toxicity in 18% of patients including 2 deaths from GVHD in spite of dose de-escalation (102). The lack of efficacy and significant toxicity seen in this small cohort of AML patients tempers enthusiasm for post-transplant PD-1 blockade, although additional studies are needed to define whether specific toxicity mitigation strategies could make this a tolerable treatment option. In addition to T cell checkpoint inhibitors, a recent addition to the armamentarium is the macrophage checkpoint inhibitor magrolimab, which targets CD47, and has shown promising activity in combination with azacitidine in newly diagnosed AML patients who are unfit for intensive therapy with an objective response rate of 71% in TP53-mutated AML (103). As detailed previously, the presence of a TP53 mutation confers an extremely poor prognosis even among AML patients who undergo allo-HCT, so it is hopeful that magrolimab’s promising activity in the newly diagnosed setting will translate to post-transplant, relapsed patients. Overall, studies of immune checkpoint inhibition for post-allo-HCT relapse have demonstrated significant toxicity with encouraging results restricted to AML patients with extramedullary relapse who were treated with ipilimumab.

### DARTs

Flotetuzumab, a CD123 x CD3 DART, represents another promising immunotherapy for the treatment of TP53-mutated AML. In a phase I/II trial, 47% of patients with relapsed/refractory AML and TP53 abnormalities had a complete response to flotetuzumab with responders demonstrating a significantly higher baseline tumor inflammation score than nonresponders. (104) While this study did not enroll patients with post-allo-HCT relapse, a study of flotetuzumab in combination with DLI is currently recruiting (NCT04582864).

### CAR T Cells

CAR T cells are approved for the treatment of relapsed/refractory B-cell acute lymphoblastic leukemia (ALL) and represent a promising modality for the treatment of post-transplant, relapsed AML. Among patients with ALL, response rates to CD19-targeted CAR T cells and 12-month overall survival are similar regardless of prior receipt of allo-HCT (105). Importantly, the comparable survival outcomes in adult patients regardless of prior transplant status suggests that prior allo-HCT does not potentiate the risk of mortal CAR T-cell toxicities. For relapsed AML after allo-HCT, CD19 CAR T cells have proven effective in a number of patients with t(8;21), which often aberrantly expresses CD19 (106, 107). While CD19 is rarely expressed in AML cells, CAR T cells targeting CD33; CD38; and CD123 have demonstrated efficacy for the treatment of post-allo-HCT AML relapse (108, 109), while CAR T cells targeting CLL1; CD13; and TIM3 among others are in development for the treatment of relapsed/refractory AML (110, 111). Thus the early evidence points to the relative safety of CAR T cells for the treatment of relapsed ALL after allo-HCT, and early studies treating relapsed AML after allo-HCT with CAR T cells targeting a variety of different antigens have yielded promising results.

### NK Cell Therapy

The importance of KIR mismatching in preventing AML relapse was initially reported in patients undergoing haploidentical allo-HCT (59), but more recent studies also point to the importance of KIR mismatching following fully matched transplant (112). A small study of eight patients (6 AML and 2 MDS) with post-allo-HCT relapse demonstrated that haploidentical NK cell infusion following lymphodepleting chemotherapy was safe and produced remission in 25% of patients, although the haploidentical NK cells did not persist, leading to relapse within 2 months (113). However, the pre-emptive administration of donor-derived, expanded NK cells after haploidentical transplantation has shown promise as a strategy to decrease early post-transplant relapse (114). While the GVL effect is primarily mediated by donor T lymphocytes, NK cells also play a role, and further studies to improve the persistence of allogeneic NK cells may help to produce more durable outcomes.

### Second Transplant

Another form of cellular immunotherapy for AML patients who relapse following allo-HCT is a second allo-HCT. Similar to DLI, a second allogeneic transplant produces the best outcomes when performed in remission and in patients with a longer duration of remission following their initial transplant. In an EBMT registry study including 455 patients with relapsed AML following allo-HCT who received a second transplant from a matched unrelated or haploidentical donor, the two factors that predicted poorer leukemia-free survival (LFS) following second transplant were transplant with active disease versus CR (HR 1.48, p=0.004) and duration of remission >13.2 months following the first transplant (HR 0.53, p<0.0001) without appreciable differences based on donor type (115). Unsurprisingly, the clinical factors that predict outcome following a second allo-HCT are similar to those that predict outcome after DLI. A separate EBMT registry study failed to demonstrate an improvement in outcomes by switching donors for the second allo-HCT for AML (116), but a small study of 40 patients demonstrated improved overall and event-free survival in patients whose second transplant utilized a new mismatched haplotype. While the authors attempted to determine whether acquired uniparental disomy (UPD) of chromosome 6p as a mechanism of genomic HLA loss played a role in relapse for patients who had previously undergone haploidentical transplant, the small patient numbers precluded any definitive conclusions (117). This well characterized mechanism of immune evasion in patients who have undergone haploidentical transplant provides a reasonable rationale for choosing 2^nd^ transplant over DLI as consolidation therapy in certain patients. However, there is a lack of compelling data to guide the choice between these two options as consolidative cellular immunotherapy in most AML patients with post-transplant relapse. A retrospective study of 418 patients from the EBMT attempted to compare outcomes with second transplant versus DLI for AML and demonstrated similar 5-year overall survival (19% for 2^nd^ transplant vs. 15% for DLI), although patients receiving 2^nd^ transplant were significantly younger (median age 43 vs. 49), more likely to have achieved remission prior to cellular therapy (38.7% vs. 18.1%), and had a significantly longer interval from 1^st^ transplant to relapse (median 348 days vs. 211 days). Notably, the incidence of NRM at 2 years was significantly higher among patients who received second transplant (26% vs. 9%) as was the incidence of grade 2-4 aGVHD at day +100 after intervention (37% vs. 20%), but there were no differences in the incidence of cGVHD (118). Based on these results, DLI and 2^nd^ allo-HCT are both reasonable options as consolidation for patients achieving remission, but 2^nd^ transplant clearly carries an increased risk of toxicity.

## Discussion

**Figure 1:**
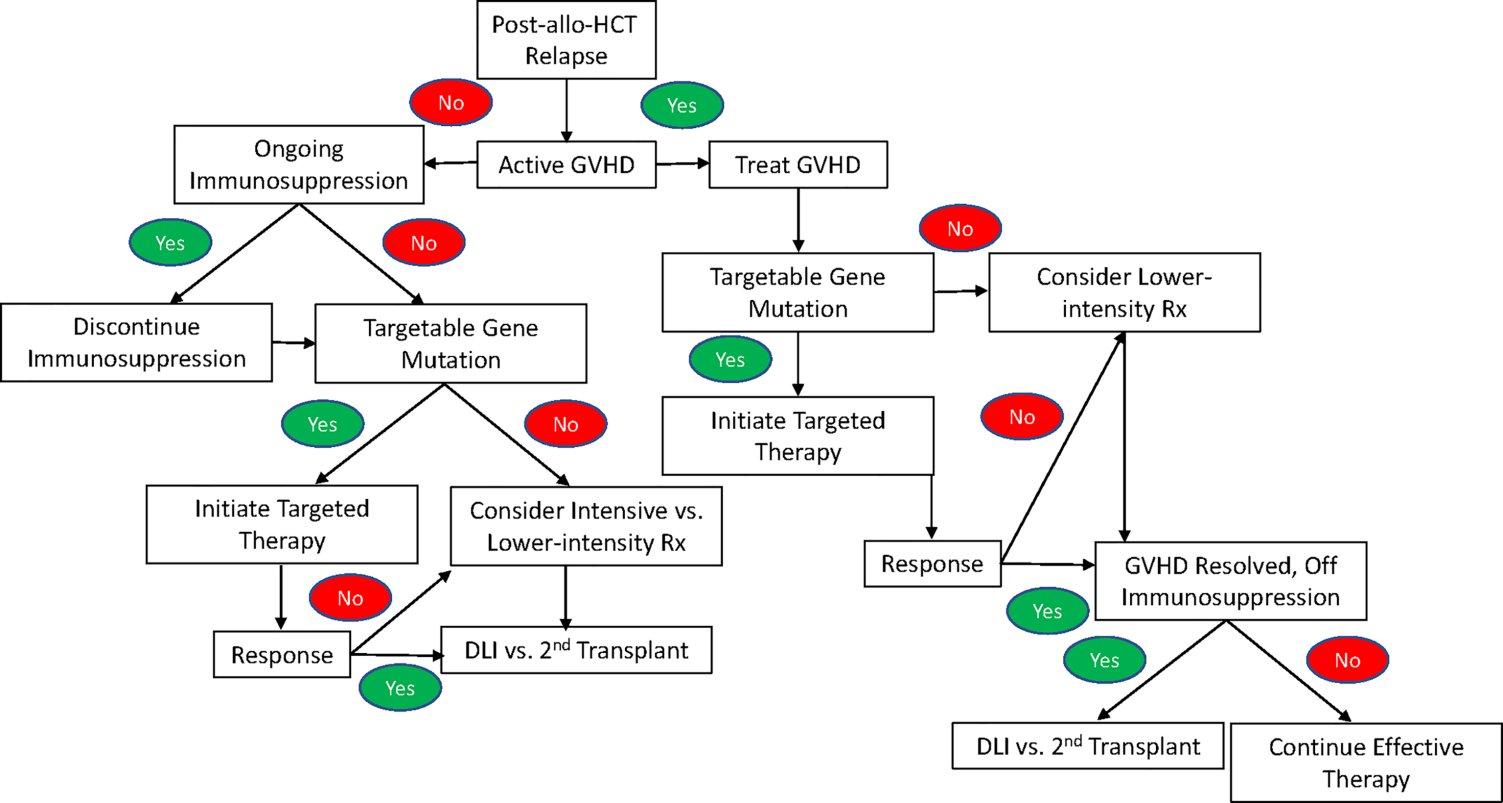
An algorithm for the treatment of relapse of AML after allogeneic hematopoietic stem cell transplant following a standard-of-care approach in the absence of an available clinical trial.

AML relapse following allo-HCT is a common problem, and its management presents additional challenges compared to relapsed/refractory AML in the non-transplant setting due to potential for residual complications from allo-HCT, especially GVHD. While randomized trials are lacking, treatment seems to be most effective when initiated prior to frank morphologic relapse when falling donor chimerism or recurrent leukemia-defining mutations suggestive of measurable residual disease (MRD) are detected. (44). Another area of active investigation is the use of maintenance therapies to prevent post-transplant relapse. This review has primarily focused on options for the management of frank morphologic relapse. As illustrated in **Figure 1**, treatment for patients with ongoing or unresolved complications from GVHD at the time of relapse necessarily involves the ongoing management of GVHD, which may limit options for further therapy, particularly due to the potential for intensive therapies to increase toxicity. These patients are also often excluded from clinical trials due to the need for ongoing immunosuppression and their increased risk of complications. Patients with active GVHD may be candidates for targeted therapies against FLT3; IDH1; and IDH2, as there is not currently evidence to suggest that these therapies are likely to exacerbate GVHD. If GVHD can be controlled, then lower-intensity therapies such as azacitidine, decitabine, or azacitidine/venetoclax may be an option for those without targetable mutations or who fail to respond to targeted therapy. Ultimately, any treatment responses achieved with targeted or lower-intensity therapies will not be durable, so subsequent cellular immunotherapy with DLI or a 2^nd^ allogeneic transplant should be considered. However, this must be approached with caution, as a history of grade 2-4 acute GVHD prior to a second transplant or DLI has been shown to lead to significantly increased NRM and poorer survival compared to patients without such a history (118).

In patients without active or unresolved complications of GVHD who suffer a post-transplant relapse, treatment begins with the withdrawal of immunosuppression. However, this is rarely an effective strategy in isolation, so additional treatment is indicated. Given the lack of a standard-of-care approach in patients with post-allo-HCT relapse, enrollment on a clinical trial is preferred. **Figure 1** provides an algorithm for treatment following a standard-of-care approach when a clinical trial is not available. While there are no trials comparing targeted therapy to intensive therapy or lower-intensity treatment specifically in the post-allo-HCT relapse setting, the available data for FLT3, IDH1, and IDH2 inhibitors suggest comparable or improved response rates with decreased toxicity, making targeted therapy the treatment of choice for relapsed AML with a targetable mutation. In patients who lack a targetable mutation or fail to respond to targeted therapy, a number of factors guide the choice between intensive and lower-intensity therapy including patient age, performance status, co-morbidities, and prior lines of therapy. Additionally, poor outcomes with intensive therapy among patients with an early post-transplant relapse (i.e. <6 months) often make it difficult to justify the potential for added toxicity, except in fit, young patients and those with rapidly proliferative disease that cannot be adequately controlled with lower-intensity therapy.

If a response is achieved following intensive; lower-intensity; or targeted therapy, then subsequent cellular immunotherapy with DLI or a 2^nd^ transplant is indicated, as there is not currently any data to suggest that such responses are durable in the absence of cellular immunotherapy. For patients with persistent leukemia despite treatment of post-transplant relapse and reasonable fitness, DLI or a second transplant with active disease still may be an option given a 5-year survival of 11% with either therapy in this scenario (118). However, other factors that influence the likelihood of successful cellular immunotherapy, such as the time from first transplant to relapse, must be carefully considered in such patients due to the significant potential for toxicity.

Ultimately, the recent proliferation of novel agents for the treatment of AML including both targeted therapies and immunotherapies, which may both enhance the GVL effect, is altering the landscape for treatment of post-allo-HCT AML relapse. This review highlights the unmet need for further studies in this area, especially as increases in the frequency of allo-HCT for AML coupled with decreases in NRM due to improvements in supportive care and the use of reduced-intensity conditioning place more patients at risk for post-transplant relapse. As we continue to better understand the immunologic mechanisms underlying the GVL effect, some of these novel therapies may serve to enhance it without exacerbating GVHD. Furthermore, a better understanding of the immunologic mechanisms underlying post-transplant relapse may facilitate the rational selection of treatment strategies to overcome those mechanisms such as 2^nd^ transplant with haplotype switch for patients who have relapsed after haploidentical transplant with leukemic blasts demonstrating loss of the mismatched haplotype. Thus advancements in the laboratory may help to guide our use of the expanding options for the treatment of post-allo-HCT relapse of AML.

## Author Contributions

JW and IG contributed equally to the conception of the manuscript. JW drafted the initial manuscript. IG and LL revised the initial draft. JW, LL, and IG agreed upon the final contents of the manuscript. All authors contributed to the article and approved the submitted version.

## Funding

This work was supported by NIH grant P30 CA06973.

## Conflict of Interest

The authors declare that the research was conducted in the absence of any commercial or financial relationships that could be construed as a potential conflict of interest.

## Publisher’s Note

All claims expressed in this article are solely those of the authors and do not necessarily represent those of their affiliated organizations, or those of the publisher, the editors and the reviewers. Any product that may be evaluated in this article, or claim that may be made by its manufacturer, is not guaranteed or endorsed by the publisher.
